# Would program performance indicators and a nationally coordinated response accelerate the elimination of tuberculosis in Canada?

**DOI:** 10.17269/s41997-018-0106-x

**Published:** 2018-07-16

**Authors:** Courtney Heffernan, Richard Long

**Affiliations:** 1 Tuberculosis Program Evaluation and Research Unit, Department of Medicine, University of Alberta, 8333 Aberhart Centre, 11402 University Avenue NW, Edmonton, Alberta T6G 2J3 Canada; 2 School of Public Health, University of Alberta, Edmonton, Alberta T6G 1C9 Canada

**Keywords:** Tuberculosis elimination, Public health, Performance indicators, Élimination de la tuberculose, Santé publique, Suivi des résultats

## Abstract

Twenty years ago, a National Consensus Conference on Tuberculosis (TB) recommended that the provinces and territories of Canada jointly declare a commitment to TB elimination with national coordination and assured funding, executed by a committee of federal and provincial/territorial representatives. Canada has committed to the global TB elimination targets set forth by the World Health Organization but lacks a coordinated response. In particular, with the exception of one published and implemented by Indigenous Services Canada, there has been no national monitoring and performance framework. Herein, we provide a commentary on the importance, to TB elimination in Canada, of developing such a framework. We invite a debate about whether more can and should be done to *monitor and report for action* at every jurisdictional level. Of utmost importance will be the need to achieve consensus from stakeholders about what is measured, among whom, how often, who collects and processes data, and how to respond to the successes and failures those data indicate. Insofar, as performance targets are well defined and implemented, national progress towards tuberculosis elimination should accelerate.

## Résumé

Il y a 20 ans, la Conférence nationale de concertation sur la tuberculose recommandait aux provinces et aux territoires du Canada de s’engager conjointement à mettre en œuvre un programme d’élimination de la tuberculose coordonné à l’échelle nationale et jouissant d’un financement garanti, exécuté par un comité de représentants des autorités fédérales, provinciales et territoriales. Le Canada souscrit aux cibles mondiales d’élimination de la tuberculose établies par l’Organisation mondiale de la santé, mais manque une réponse coordonnée. En particulier, sauf pour celui publié et mis en œuvre par Services aux Autochtones Canada, il n’y a aucun cadre national de suivi des résultats. Notre commentaire porte sur l’importance d’un tel cadre pour l’élimination de la tuberculose au Canada. Nous cherchons à engager un débat pour déterminer s’il est possible et souhaitable d’en faire plus pour assurer *le suivi et le compte rendu des mesures prises* à tout ordre de gouvernement. Il sera de la plus haute importance de parvenir à un consensus des acteurs sur ce qui est mesuré, par qui, à quelle fréquence, qui recueille et traite les données, et comment réagir aux réussites et aux échecs que ces données indiquent. Dans la mesure où les cibles de résultats sont bien définies et mises en œuvre, les progrès réalisés à l’échelle nationale en vue d’éliminer la tuberculose devraient s’accélérer.

## Introduction

As a co-signatory to the World Health Organization End TB Strategy, Canada has a responsibility to meet certain country-specific TB elimination targets: “pre-elimination”—reaching 10 cases per million by 2035, and “elimination”—reaching 1 case per million by 2050 (The World Health Organization (WHO) 2014). Unfortunately, with the disease now confined, almost exclusively, to Indigenous peoples and foreign-born migrants who are poorly positioned to advocate for themselves, and the rate having stayed between 4 and 5 per 100,000 over the past 15 years, the chances of meeting these targets, without major programmatic changes, are virtually impossible.

Directives aimed at TB elimination are not new to Canada. Twenty years ago, a National Consensus Conference on TB recommended that the provinces and territories jointly declare a commitment to TB elimination with national coordination and assured funding, executed by a committee of federal and provincial/territorial representatives (Proceedings of the National Consensus Conference on Tuberculosis 1998). Collaboration across jurisdictions with a “national dimension” of monitoring has been implemented successfully elsewhere, notably in the United States (US); unfortunately, despite the recommendations of the consensus conference, it is still missing from Canada two decades later (Centers for Disease Control (CDC) and Prevention 2012; Lönnroth et al. 2015). A 2016 *Blueprint for a Federated System of Public Health Surveillance in Canada* published by the Pan-Canadian Public Health Network provides additional  support in favour of, and a recent justification for, federally supported surveillance efforts and a framework for a way forward; as such, we should consider ourselves ready to act (Pan-Canadian Public Health Network 2016). In the following commentary, we provide a rationale for a TB-specific monitoring and performance framework, report on the scale of the challenge, and introduce the concept of program performance indicators applicable to Canada as a whole, its provinces, territories, and population groups.

### Rationale

Canada is a large country with a relatively small population concentrated in its southern latitudes. Most (> 90%) cases of TB are among Indigenous peoples and the foreign-born (Tuberculosis in Canada 2016 Pre-release. Public Health Agency of Canada. Minister of Public Works and Government Services Canada 2018; Long et al. 2013). With respect to Indigenous cases, a south-north gradient of risk has emerged with the highest rates and greatest burden of disease now reported in the north (Long et al. 2013). With respect to the foreign-born cases, most are reported in major metropolitan areas. The TB stakeholder community is not large and is mostly concentrated in the major immigrant-receiving provinces of Quebec, Ontario, Alberta, and British Columbia. These realities suggest that for Canada to achieve the ambitious TB elimination targets set out by the WHO and to which it has committed, the community of TB stakeholders, nationally, should organize to develop a network of cross-jurisdictional expertise. In other words, the resources of all stakeholders should be pooled as provinces, territories, and relevant federal departments work together in support of a country-specific strategy aimed at reaching pre-elimination and elimination targets. Provider delays in the diagnosis and treatment of TB due to a lack of access, service, or expertise are a social injustice and continue to occur in First Nations and Inuit communities (Galloway 2018).

TB is a complex disease, and its management and care cuts across many jurisdictions. It is a notifiable disease in all provinces and territories under relevant Public Health Legislation. By agreement, provincial, territorial, and federal—Indigenous Services Canada (ISC)—TB control programs report their TB notification and treatment outcome data to the Public Health Agency of Canada (PHAC) who, in turn, produce a national, surveillance snapshot using the Canadian Tuberculosis Reporting System (CTBRS) (Tuberculosis in Canada 2016 Pre-release. Public Health Agency of Canada. Minister of Public Works and Government Services Canada 2018). Most health services, including public health, are provided and funded by provinces and territories while others—for example, to registered First Nations and some Inuit—are a federal responsibility; communication across jurisdictions is usually limited. To achieve TB elimination, coordinating surveillance efforts across these jurisdictions is essential, and requires assured and sustained political and financial support from the federal government.

It is not unreasonable to conclude that the remarkable success—reporting the lowest national TB incidence among G7 nations in 2016—the US has achieved is in part attributable to the monitoring and performance framework their Centers for Disease Control (CDC) developed some years ago: the National TB Indicators Project (NTIP) (Centers for Disease Control (CDC) and Prevention 2012). The aforementioned CTBRS is, similarly, a national repository of data from provincial/territorial TB notification forms, but Canada has heretofore not availed itself of the system as an operational tool in the cause of disease elimination. Current CTBRS data are not organized in such a way as to track key indicators by jurisdiction, or population group, nor are reports from this system up to date, with the last full *Tuberculosis in Canada* report published in 2012; the last pre-release is for 2016.

### Scale of the challenge

**Fig. 1:**
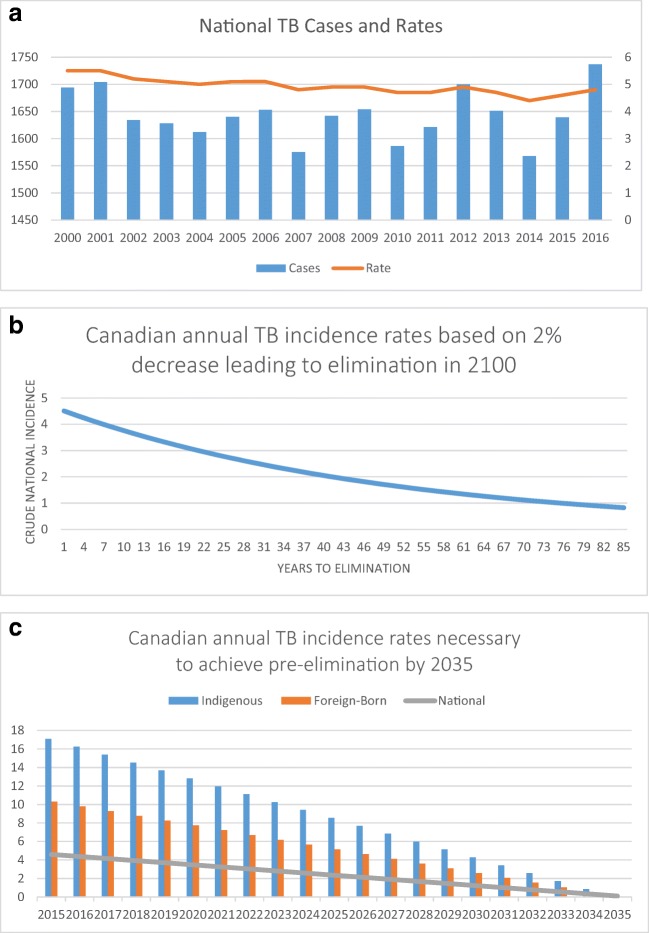
**a** National tuberculosis cases and rate of disease (2000–2016). Actual: Between 2000 and 2016, the national rate declined by 16% or ~ 1% per annum—half of the most optimistic estimates and less than a quarter of the most aggressive targets we would need to achieve in order to meet the 2035 elimination goals. **b** Estimated number of years (~ 85) to pre-elimination targets given a 2% per annum reduction in incidence. Optimistic: Based on conventional wisdom that once the foreign-born migrants contribute more than 70% of cases, the annual incidence, using routine or continued programming, will not decline by more than 2% per year. Foreign-born migrants have contributed > 70% of cases since 2015. **c** Decline in national incidence to 10/1,000,000 population by 2035. Aggressive: Ideal reduction in the annual TB incidence rate to meet pre-elimination by 2035

The WHO TB elimination targets are not likely to be achieved without significant programmatic changes. The national rate of TB disease has virtually plateaued, with a relatively minor decline in incidence from 2000 to 2016 (Fig. 1 a). It has been estimated that once the foreign-born migrants contribute 70% or more of cases within a country, which is now the case in Canada, the rate of decline is not expected to exceed 2% per year without making changes to existing programs or reducing the prevalence of disease overseas (The World Health Organization (WHO) 2008). At this rate of progress, we will not meet the internationally established, and nationally supported TB elimination targets (Fig. 1 b). To meet *pre*-elimination targets, cases among both Indigenous peoples and the foreign-born have to decline by 95%, or 4.52% per annum corresponding to annual reductions in the incidence rate of 0.855 and 0.515 per 100,000 population, respectively (Fig. 1 c). Use of granular and up-to-date program performance indicators could help to identify key “sticking points”, which programs and communities could then use to generate solutions that accelerate elimination.

We recommend one possible indicator collection and management scenario: a committee of provincial, territorial, and federal representatives is struck with the mandate of adopting the WHO post-2015 global TB strategy principle of “government stewardship and accountability [at all levels] with monitoring and evaluation” (The World Health Organization (WHO) 2018). This committee of stakeholders and partners should meet annually to interpret findings, request additional analyses as needed, and make recommendations to improve program performance in each jurisdiction. Shared experience and expertise, from the community up, is understood to be to the benefit of all. As a tool of surveillance, the CTBRS would collect and organize data for each of the suggested jurisdictions and population groups with indicators coming from or added to existing forms (Active Case Report Form; Treatment Outcome Form). Other surveillance strategies are also possible.

Data not currently reported in the CTBRS, but which were approved as indicators in the Pan-Canadian Public Health Network *Guidance for Tuberculosis Prevention and Control Programs in Canada*, the First Nations and Inuit Health Branch, *Monitoring and Surveillance Framework*, and two published Provincial program evaluations (Alberta and Manitoba) such as the results of contact investigations, would need to continue to be collected and reported in such a manner as to protect privacy and confidentiality (Proceedings of the National Consensus Conference on Tuberculosis 1998; Health Canada: First Nations and Inuit Health Branch 2016; Long et al. 2015; Basham et al. 2018). We have provided a potentially useful framework of indicators in Table 1; some indicators currently exist, while others are new. Their validity and the suggested best practice targets are recognized to be open to debate. All provinces, territories, and relevant federal ministries will need to agree, recognizing collaborative action to be in the best interest of their own jurisdiction as well as nationally. Local and provincial/territorial capacity for reporting and responding to poor performance should improve through the development of a coordinated nexus of expertise.

**Table 1:** Proposed indicators and targets

Indicator	Target
Incidence*	
• Incidence (National)	Rate of decline commensurate with 2035 pre-elimination goals
• Incidence (on- and off-reserve First Nations)	
• Incidence (Inuit Nunangat)	
• Incidence (Atlantic Canada)	
• Incidence (Quebec)	
• Incidence (Ontario)	
• Incidence (Manitoba/Saskatchewan)	
• Incidence (Alberta/Northwest Territories)	
• Incidence (British Columbia/Yukon)	
Laboratory reporting	
• Turn-around time (NAAT)	6 days (4 days specimen collection and delivery; 2 days detection)
• Turn-around time (Culture)	25 days (4 days specimen collection and delivery; 21 days to grow)—78%
• Proportion HIV tested	95%
• Proportion of culture-positive cases with DST	100%
• Proportion of culture-positive cases with genotyping	100%
for smear-positive pulmonary cases †	
• Sputum culture and CXR, end of initial phase	100%
• Sputum culture and CXR, end of continuation phase	100%
Case management and treatment	
• Proportion of pediatric cases (< 5 years)	< 5%
• CB cases/w no past hx TB started on a minimum of 3 drugs	100%
• FB cases/w no past hx TB started on a minimum of 4 drugs	100%
• Proportion with TB-related death of preceding years’ cases	< 5%
for smear-positive pulmonary cases	
• Starting treatment within 72 h of NAAT	90%
• Complete treatment within 12 months	95%
Contact investigations	
• Close contacts of smear-positive pulmonary cases:	
Proportion completely assessed < 5 years of age	100%
Proportion completely assessed ≥ 5 years of age	80%
• Close contacts with new positive TST/TST conversion:	
Proportion recommended TX LTBI < 5 years of age	100%
Proportion recommended TX LTBI ≥ 5 years of age	100%
• Close contacts recommended TX LTBI:	
Proportion who start treatment < 5 years of age	100%
Proportion who start treatment ≥ 5 years of age	80%
• Close contacts accepting TX LTBI:	
Proportion who complete treatment < 5 years of age	100%
Proportion who complete treatment ≥ 5 years of age	80%
IRCC referrals	
• Proportion who initiate examination within 30 days of notification	100%
• Proportion completing examination within 90 days of notification	100%
• Proportion of IRCC referrals recommended TX LTBI who accept	90%
• Proportion of IRCC referrals accepting TX LTBI who complete	90%
CTBRS reporting	
• Active TB case report form (last full year) complete	100%
• Treatment outcome form (next to last full year) complete	100%

*NAAT* nucleic acid amplification test, *DST* drug susceptibility testing, *CXR* chest x-ray, *CB* Canadian-born, *FB* foreign-born, *TX* treatment, *LTBI* latent tuberculosis infection, *IRCC* Immigration Refugee and Citizenship Canada, *CTBRS* Canadian Tuberculosis Reporting System

*The proposed groupings take into account persons at risk, populations, existing organizational structures, and relationships

†The initial phase of treatment refers to an intensive three-four drug treatment regimen that lasts 2 months; the continuation phase lasts 4–7 months in fully susceptible cases and is usually a two-drug regimen

The NTIP provides individualized reports back to US states for monitoring, review, and progress as well as for justifying the provision of federal support to meet objectives (Centers for Disease Control (CDC) and Prevention 2012). While national surveillance is not an intervention that can be systematically evaluated, there is strong evidence in support of individual indicators monitored by their program. The success of this US program, and proposals from other high-income, low-incidence nations to implement national TB monitoring systems, make a similar recommendation for Canada prudent.

## Discussion

Surveillance and monitoring are “disease reporting for action”—actions that are flexible and modifiable in response to what the data are portraying through an integrated program of research, which “closes the loop … so that those who report information can see that the data are being used… which, in turn, improves reporting” (Henderson 1998). Functional surveillance and monitoring is necessary to meet the needs of people everywhere in Canada who are unjustly disadvantaged by TB.

Nationally, TB disease disproportionately affects persons with limited power, status, and financial means, notably the foreign-born and Indigenous peoples (Tanday 2017). It may be that because TB affects the “voiceless”, progress towards TB elimination in Canada has been slow (Tanday 2017). Many people at risk of developing TB disease find themselves at the crossroads of multiple jurisdictions; a nationally coordinated response to disease elimination would help ensure their care meets a standard, and is not delayed. The tools and expertise are at hand to eliminate TB disease, but they need to be thoughtfully implemented in order that we get back on track. Responding to suboptimal TB program performance to get where we are going can only be achieved insofar as we know where we currently are.
